# Diversity of Rotavirus Strains in Children; Results From a Community-Based Study in Nepal

**DOI:** 10.3389/fmed.2021.712326

**Published:** 2021-10-01

**Authors:** Jasmin Shrestha, Sanjaya K. Shrestha, Tor A. Strand, Susanne Dudman, Jennifer L. Dembinski, Rose Vikse, Ashild K. Andreassen

**Affiliations:** ^1^Center for International Health, University of Bergen, Bergen, Norway; ^2^Walter Reed/AFRIMS Research Unit Nepal, Kathmandu, Nepal; ^3^Department of Research, Innlandet Hospital Trust, Lillehammer, Norway; ^4^Institute of Clinical Medicine, University of Oslo, Oslo, Norway; ^5^Department of Microbiology, Oslo University Hospital, Oslo, Norway; ^6^Department of Virology, Norwegian Institute of Public Health, Oslo, Norway

**Keywords:** rotavirus, acute gastroenteritis, community based, seasonality, genotypes

## Abstract

**Objective:** The objectives of this study were to describe the incidence and genetic diversity of Rotavirus (RV) infection among children up to 3 years of age in a community in Nepal.

**Methods:** We investigated community-acquired cases of asymptomatic and symptomatic RV infections in children from birth to 36 months of age in a community-based birth cohort in Bhaktapur, Nepal. Monthly surveillance and diarrheal stool samples were collected from 240 children enrolled at birth, of which 238 completed the 3 years of follow-up. Samples were screened for rotavirus by Enzyme Immuno Assay (EIA). All RV screened positives were further genotyped by reverse transcription-polymerase chain reaction for the capsid genes VP7 and VP4.

**Results:** In total, 5,224 stool samples were collected from 238 children, followed from birth to 36 months of age. Diarrhea occurred in 92.4% (230/238) of all children in the cohort. During the 3 years study period, RV was more frequently seen in children with symptoms (7.6%) than in non-symptomatic children (0.8%). The highest RV detection rate was found in younger children between 3 and 21 months of age. Although rotavirus is known as winter diarrhea, it was detected throughout the year except in August. The highest positivity rate was observed in the months between December and March, with a peak in January. Four common G types were seen: G2 (30%), G1 (29%), G12 (19%), and G9 (16%). The most predominant genotypes seen were G2P[4] (30%), followed by G1P[8] (27.0%), G12P[6] (14.0%), G9P[8] (10%), and remaining were mixed, partial, and untyped.

**Conclusion:** Our study confirms that rotavirus is a common cause of gastroenteritis in young children in the community. The prevalence and pathogenicity of rotavirus infection differed by age. There was substantial variability in circulating strains in the community samples compared to samples collected from hospitals. This shows the importance of including community-based surveillance systems to monitor the diversity of circulating rotavirus strains in Nepal.

## Introduction

Rotavirus (RV), a member of family Reoviridae, is the most common cause of diarrhea among children under 5 years of age globally and it is responsible for more than 200,000 deaths annually. In fact, rotavirus diarrhea accounts for 49% of all diarrheal deaths in Africa and Asia (1). By the age of five, every child around the globe has experienced at least one episode of RV diarrhea.

RV is double stranded RNA virus with two outer capsid proteins, VP4 (P serotype, for protease sensitive protein) and VP7 (G serotype, for glycoprotein) both of which are involved in virus neutralization (2–5). Both VP4 and VP7 proteins are encoded by separate gene segments. RV can generate new G-P serotype antigen combinations through reassortment and both serotype antigen are believed to be crucial for developing protective immunity. Therefore, it is important to have knowledge on and the genotype variation for both G and P types (6). Globally, rotavirus strains G1P[8], G2P[4], G3P[8], and G9P[8] are responsible for more than 90% of rotavirus gastroenteritis cases in young children (7, 8). G1P[8] was dominating in North America, Europe and Australia occurring in more than 70% of the RV infections, compared to 30% of the infections in South America and Asia, and 23% in Africa. Other common genotypes of RV infections worldwide were G3P[8], G2P[4], and G4P[8], which together with G1P[8], accounted for 50% of cases in Africa and 90% in Europe, North America, and Australia (9). In addition, G9 combination with P[8]/P[4]/P[6] were also identified together with increased incidence of G12 strains (9–11).

Two live, attenuated, oral rotavirus vaccines are currently routinely used globally: the Rotarix monovalent (RV1; GlaxoSmithKline, Belgium) and the RotaTeq pentavalent (RV5; Merck, USA). They were developed from G1P[8] and genotypes G1–G4 in combination with P[8][5], respectively. Two other vaccines have been sponsored by the government of India, the monovalent ROTAVAC-G9P[11] and the multivalent Rotasil (G1, G2, G3, G4, G9 and P[8]). Currently, these two latter vaccines are routinely administered to children in 12 Indian states (12). A wide range of circulating strains exists, including uncommon rotavirus strains reported from different parts of the world. It is important to consider these when implementing a vaccine in national vaccination programs to ensure broad cross protection against all circulating genotypes.

The incidence of rotavirus diarrhea is similar in developed and developing countries suggesting that improvement in sanitation and drinking water facilities is not sufficient to prevent transmission of RV (13). However, the case fatalities are much higher in poor vs. affluent populations which is likely because of limited access to health care, lack of available hydration therapy and a greater prevalence of comorbid conditions (such as malnutrition), among other factors (14). This poverty-related severity and mortality was observed before the introduction of vaccines. The results from clinical trials show lower efficacy of RV vaccines in marginalized compared to affluent settings. Nevertheless the implementation of rotavirus vaccines in national immunization programs has substantially reduced the disease burden of RV infections (15). WHO has recommended the rotavirus vaccine in immunization programs globally, particularly in countries with high RV lethality in children under 5 years of age (16). Rotavirus vaccination (Rotarix) was recently (July 2020) introduced in the national immunization program in Nepal.

In Nepal, hospital-based studies conducted in 1999–2014 showed infection rates ranging from 17 to 39% among children <5 years (17, 18). The most common G-types were G12, G1, G2, G9 in children with diarrhea. The hospital-based studies describe the most severe acute gastroenteritis cases and might not be representative for the burden of acute gastroenteritis in the general population. The present study investigates community acquired cases of asymptomatic and symptomatic rotavirus infections in different age groups of children in the pre vaccination era in Nepal. Moreover, the study also describes the genotype distribution of rotavirus circulating in populations of children with and without symptoms of gastroenteritis in Nepal.

## Materials and Methods

### Study Site, Population, and Sampling

This is a descriptive study and includes stool samples collected between June 2010 to February 2015 from children enrolled in the birth cohort “Malnutrition due to Enteric Diseases (MAL-ED)” study. The study was carried out in Bhaktapur, Nepal, which is a semiurban area 12 km from the capital Kathmandu. During the MAL-ED study period, 240 newborn children were recruited within 17 days after birth and each child were visited biweekly. Structured questionnaires for caregiver were provide to collect clinical information of child with anthropometric measurement and periodic sample collection from the time of enrollment, during each diarrhea episode upto 36 months of age, continuously. Subject enrollment occurred over a 2 years period, the earliest enrollment was initiated in June 2010 and the latest enrollment occurred in February 2012. Details of study design, sample collection, and microbiological methods have been published previously (19, 20). Briefly, surveillance stool (non-diarrheal) samples were collected monthly for the first 12 months and then quarterly upto 36 months. All diarrheal episode stool samples were collected from enrolled children. A total of 5,224 stool samples were collected and were analyzed for the presence of bacteria, viral, and parasitic pathogens associated with diarrhea using traditional methods of microscopy, culture, enzyme-linked immunosorbent assay (ELISA), and polymerase chain reaction (PCR) as appropriate to pathogens. Bacteria (*E. coli, Salmonella* spp., *Shigella* spp., *Vibrio* spp., *Aeromonas* spp., *Yersinia* spp., and *Plesiomonas* spp.) were identified by conventional culture methods, virus (Rotavirus, Adenovirus, Astrovirus) and parasites (Giardia, Cryptosporidium) by Enzyme Immuno Assay (EIA). Diarrheagenic *E. coli* [enteropathogenic, enterotoxigenic, enteroaggregative, Shiga toxin (1 and 2)–producing, and enteroinvasive strains] and Norovirus (GI and GII) were identified using PCR. Excess stool samples were archived for future analysis.

### Laboratory Testing

The collected stool samples were screened for rotavirus antigen by the VP6-ELISA assay (ProSpect TM Rotavirus microplate assay, Oxoid Ltd., UK). Further genetic characterization was performed using the frozen stool samples stored from children with rotavirus antigen. In total, nine frozen stool samples positive for RV antigen had insufficient volume and were excluded for genetic characterization. The samples were coded with numbers to delink them from personal identity.

Ethical approvals were received from the ethical board, Nepal Health Research council (NHRC), National IRB of Nepal, and the Norweigen regional ethical committee for medical and health research (REK sør-øst B 42335, Norway). A written informed consent was obtained from the caretaker for all participants in the study.

### Rotavirus Genotyping

Extraction of the viral RNA from available EIA RV positive stool samples was done by the QIAmp RNA Mini Kit (Qiagen, Hilden, Germany). Detection of rotaviruses in clinical specimens by reverse transcription polymerase chain reaction (RT-PCR) of the G-type and P-type was accomplished by following standard methods according to Gunasena et al. (5) and Vainio et al. (21). All samples were amplified with primers specific for the VP7 genes of G serotypes 1, 2, 3, 4, 8, 9, 10, and 12 and VP4 genes of P serotypes 4, 6, 8, 9, 10, and 11 as described by Gouvea (22, 23). Amplified amplicons were separated into different bands size genotypes by gel electrophoresis as described by Gouvea et al. (22) and Gentsch et al. (24) with further modification made by Iturriza-Gómara et al. (25). RNA positive control and Negative controls were added in the PCR assay for validation.

### Data Analysis

The prevalences of RV was calculated by dividing numbers of cases by total number of study sample collected during the study period. We calculated the risk of being rotavirus positive in diarrhea and non-diarrhea stool samples. The risk ratio (RR) and corresponding 95% confidence intervals (CI) between diarrhea and non-diarrheal stool samples for three age groups of children were calculated using descriptive statistical method (Microsoft Excel version 2016). Statistical tests for between-group comparisons were based on Fisher Exact test (Microsoft Excel Version 2016). A significance level of *p* < 0.05 was used for all statistical analysis.

## Results

### Epidemiology of Rotavirus

A total of 5,224 stool samples were collected from 240 children in the community, of these 1,160 were diarrheal stool samples and 4,064 were non-diarrheal stool samples. Out of 240 enrolled children, 238 children completed 36 months of follow up. The mean age of the children experiencing diarrhea with rotavirus infection was 13.6 months (SD ± 6.67 months) with a median age of 14 months. A total of 119 samples (2.28%) were positive for rotavirus by EIA. From the cohort 95.8% (230/240) of the children experienced episodes of diarrhea. During the 3 years period, rotavirus was more frequently detected in the diarrheal samples (88/1,160 [7.6%]) than in the non-diarrheal samples (31/4,064 [0.8%]) RR = 10.7; 95% confidence interval (CI), [7.1, 16.2], *p* < 0.001. When stratified by age group, children <25 months of age were at a higher risk of rotavirus infection as compared to older children, and the risk of being infected decreased with increasing age in both the diarrheal and non-diarrheal groups (Table 1).

**Table 1:** Rotavirus detection by enzyme immunoassay (EIA) in different age group of 240 Nepalese children.

**Age group**	**Rotavirus positive** (***N***)		**Rotavirus negative** (***N***)		**RR**	**95% CI**	***P*** **-value**
	**Diarrhea**	**Non-diarrhea**	**Diarrhea**	**Non-diarrhea**			
0–12	43	28	539	2,674	7.13	[4.47, 11.38]	<0.0001
13–25	42	1	405	886	83.34	[11.51, 603.59]	<0.0001
26–36	3	2	128	473	5.44	[0.92, 32.21]	0.062

*RR, Risk Ratio; N, total number of samples tested*.

### Distribution of Rotavirus Genotypes

**Figure 1:**
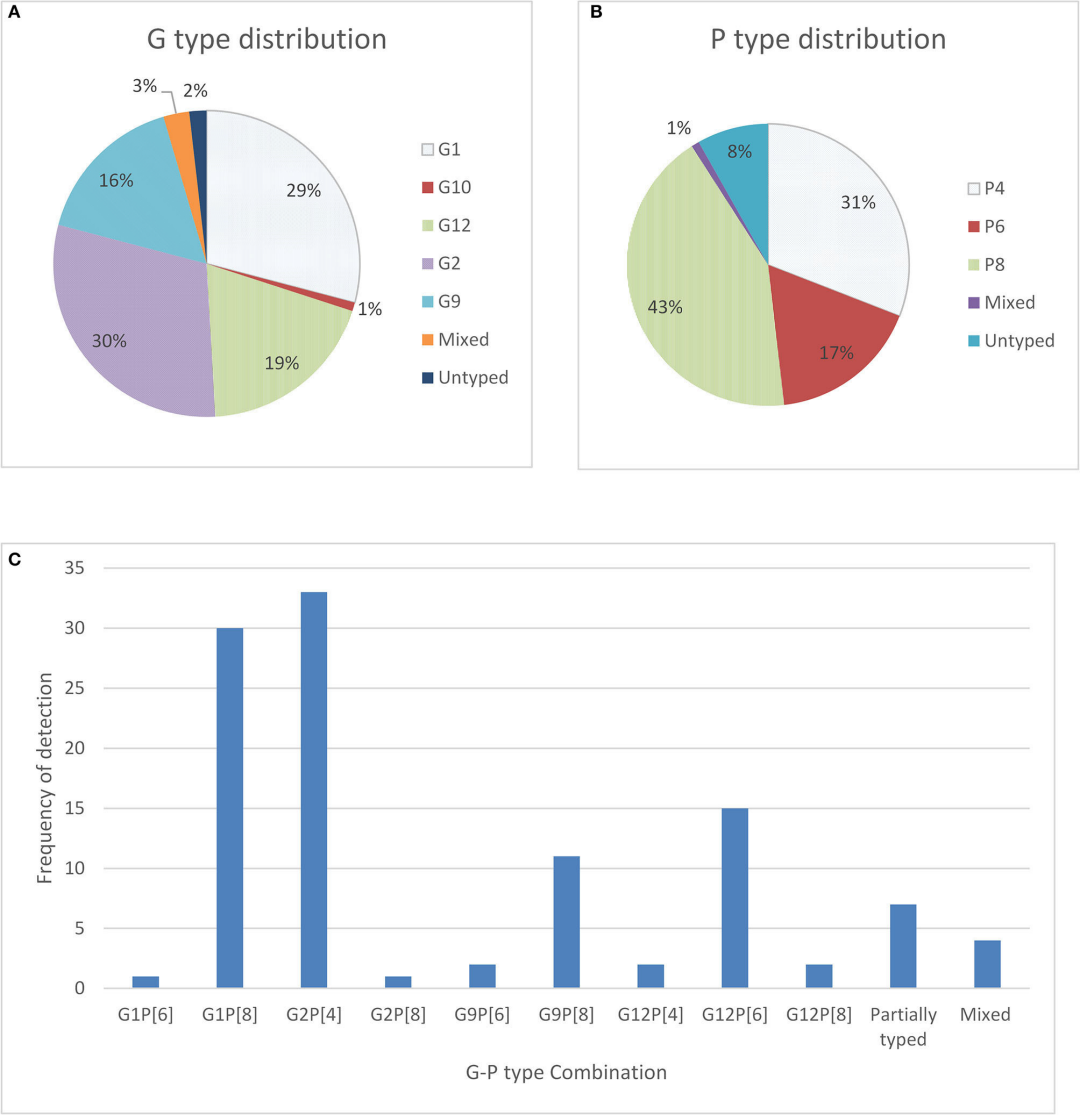
Distribution of G types and P types; **(A)** total percentage of different G types detected in children with symptoms as well as without symptoms **(B)** total percentage of P types rotavirus strain detected among children with symptoms as well as without symptoms and **(C)** frequency and cumulative percentage of G and P genotypes of rotavirus stains detected among community children.

In our study, only 110 frozen stool samples, confirmed to be positive for RV antigen by EIA were available for genotyping. One hundred and eight (98%) of those stool samples were able to be characterized for genotype, 108 were positive for G types, 101 were positive for P types, and 2 were untypable or not typable (NT) for both P and G types. The most dominating G type was identified as G2 (30%), followed by G1 (29%), G12 (19%), G9 (16%) and the remaining were mixed types including G10. For P types, P[8] (43%) was found most frequently, followed by P[4] (31%) and P[6] (17%) (Figure 1). In seven stool samples no P types could be assigned.

**Figure 2:**
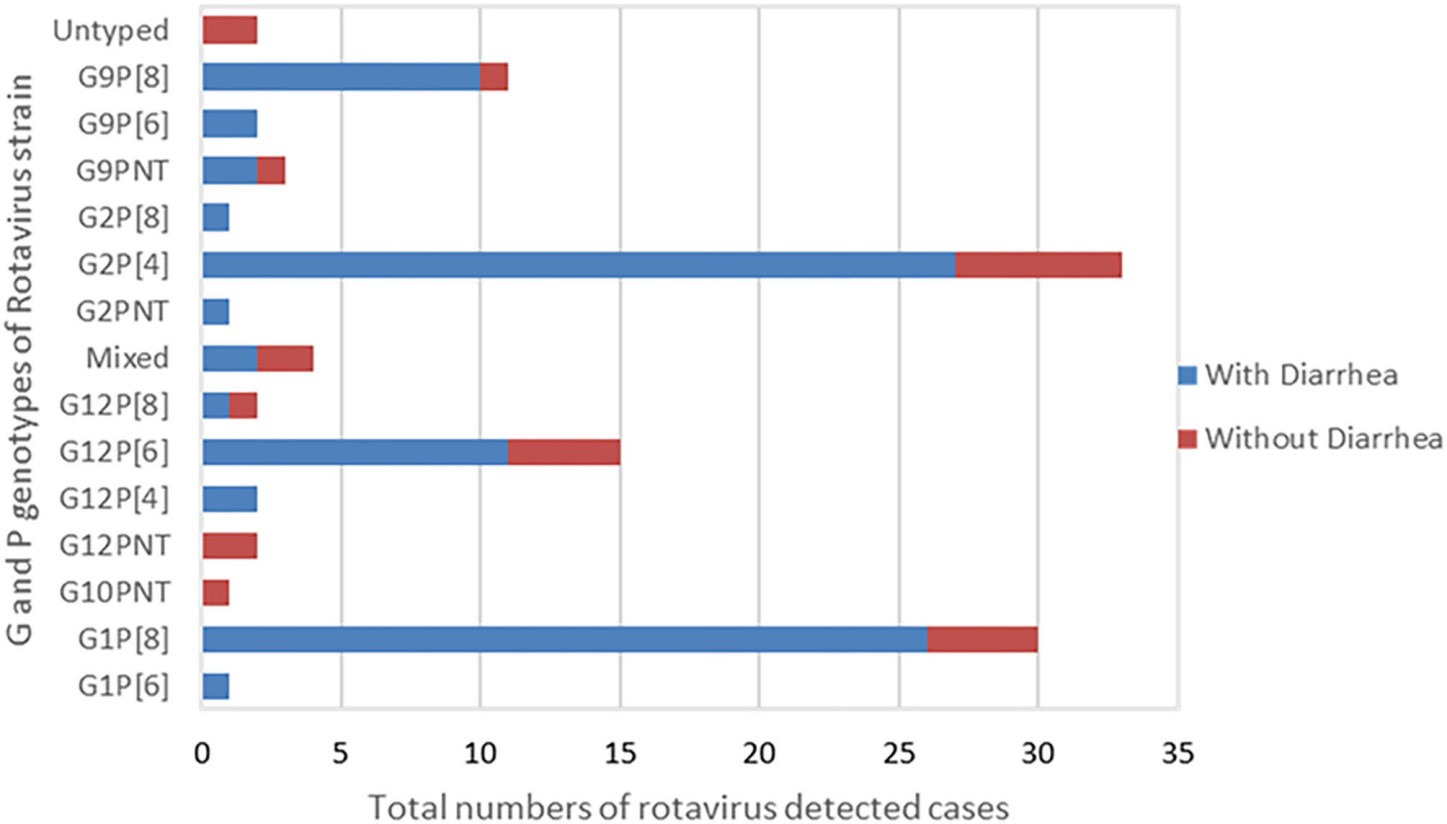
G and P Genotype combination of the rotavirus strains typed by RT-PCR. The graph presents total numbers of rotavirus strain of G and P genotype combination detected in children with presentation of diarrhea and without diarrhea.

The most predominant G and P type combination were Genotype G2P[4] (30%) followed by G1P[8] (27.0%), G12P[6] (14.0%), and G9P[8] (10%). Others genotypes such as G1P[6], G12P[4], G12P[8], G2P[8], and G9P[6] were seen in lower proportions (Figure 1). Mixed strains such as G1G2P[8], G1G9G12P[6], G1G9P[8], and G9P[4,6] were found in four samples. Partially typed rotavirus strains such as G12 and G10 with un-typable P type and mixed type G1G2P[8] and G1G9G12P[6] were found in non-diarrheal samples (Figure 2).

G12 and G9 strains were seen in combination with P[4], P[6], and P[8] and comprised 17% (14/82) each of the total genotypes in children with diarrheal episode (Figure 2).

### Distribution of Rotavirus Genotypes by Age Group

**Figure 3:**
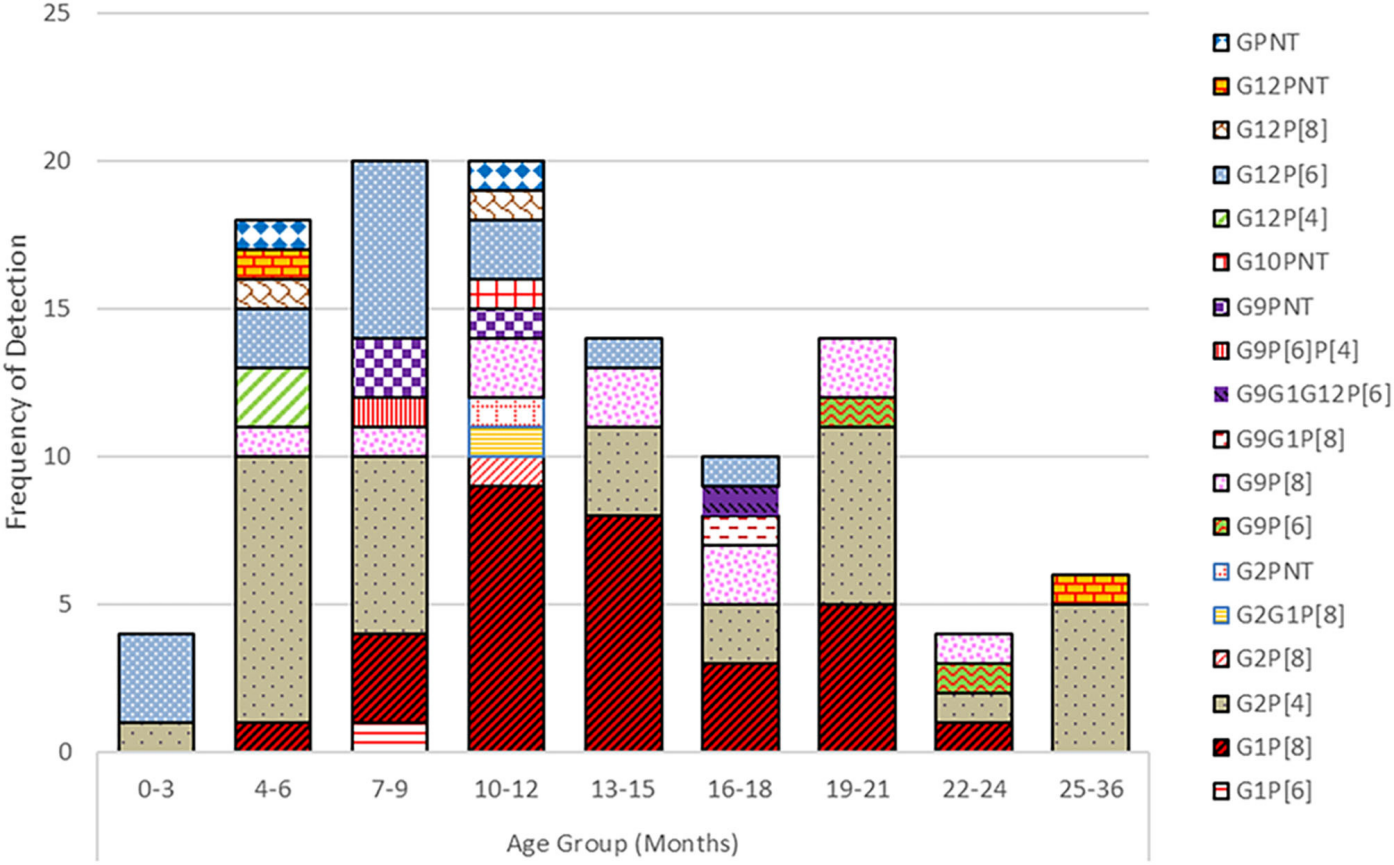
Prevalence of G and P genotypes combination of the rotavirus strains detected in different age groups of children.

Rotavirus was primarily detected in children below 2 years of age, most frequently affecting the 7–24 months age group (Figure 3). Infants from 0 to 12 months contributed with 45% of the rotavirus associated diarrhea whereas 50% of cases were seen in the 13–24 months age group and in 5% of the 2 years or older group. Infants ≤3 months of age contributed 2% of diarrhea cases associated with rotavirus. The G2P[8] strain was detected in all age groups of children whereas G12P[6] was found in younger children below 18 months of age. G9P[8] and G1P[8] genotypes were detected only in children above 3 months of age till 2 years of life. In 4–12 months, old children, seven to nine different rotavirus genotypes were found.

### Seasonal Distribution of Rotavirus

**Figure 4:**
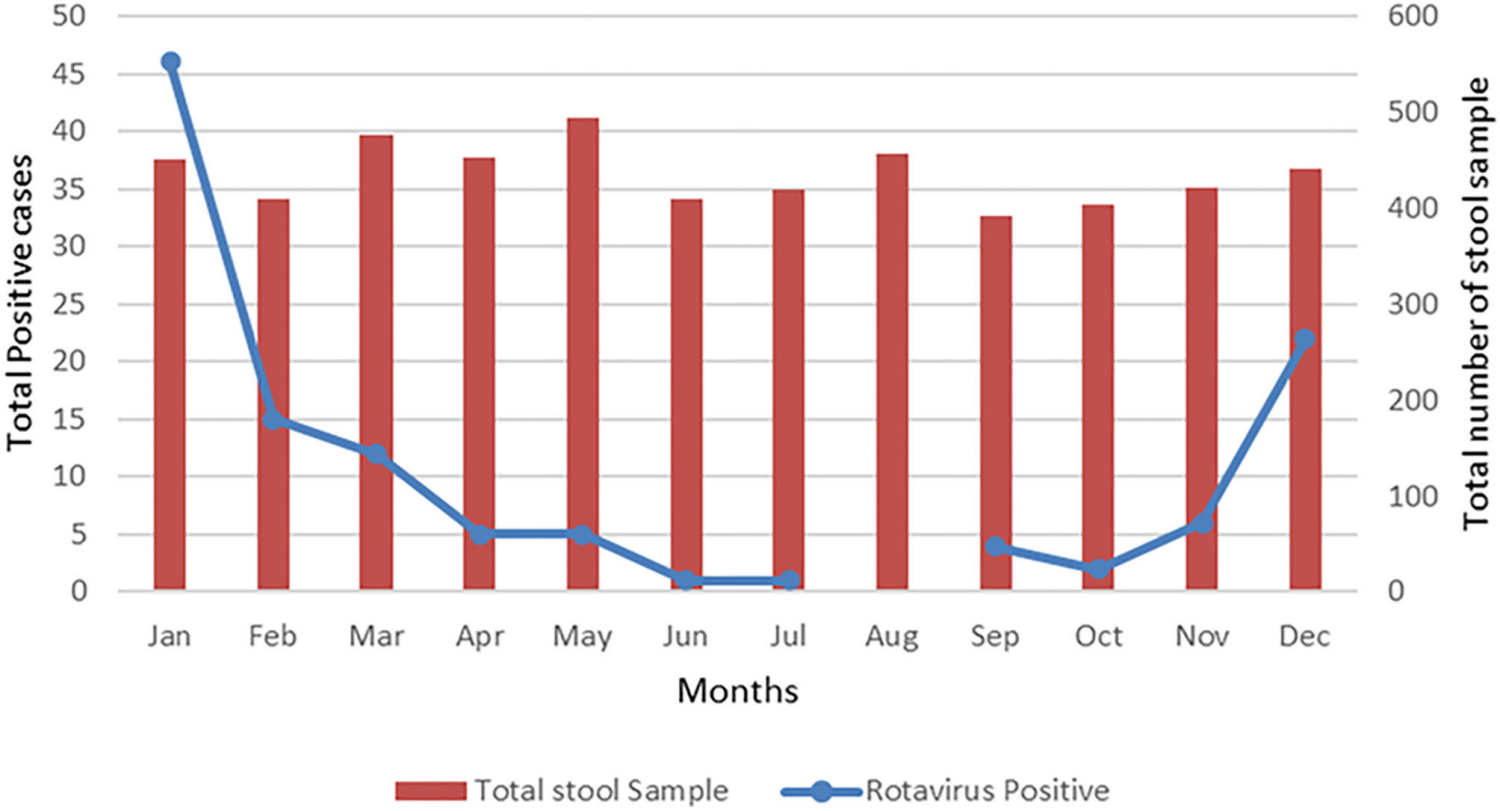
Seasonal variation of rotavirus infection among cohort children. The graph shows the total number of rotavirus infected in different months (blue dots, left axis) of total number of samples collected (red bars, right axis).

RV infection was most frequently observed in the winter months between December and March with the highest peak occurring in January, which is the coldest month of the year in Nepal (Figure 4). No cases were detected in the month of August.

### Coinfection of Rotavirus With Other Pathogens

RV was seen co-infecting with bacterial, virus and parasites in 55.5% (66/119) of stool samples. Bacterial coinfection was observed in 38% of stool samples. RV was detected together with *Campylobacter* in 21 samples and with diarrheagenic *E.coli* (EAEC and EPEC) in 38 samples. Details of coinfections are shown in Table 2.

**Table 2:** Coinfection of rotavirus with other enteric pathogens.

**Pathogens**	**No. of detection**
**1. Single pathogen**	
Rotavirus	53
**2. Pathogens**	
Adenovirus, Rotavirus	3
Astrovirus, Rotavirus	3
Norovirus GII, Rotavirus	2
*Campylobacter*, Rotavirus	14
EAEC, Rotavirus	18
EPEC, Rotavirus	7
Giardia, Rotavirus	4
Cryptosporidium, Rotavirus	2
**3. Pathogens**	
EAEC, Adenovirus, Rotavirus	1
EAEC, Norovirus GII, Rotavirus	1
*Campylobacter*, EAEC, Rotavirus	5
*Campylobacter*, EPEC, Rotavirus	1
EAEC, *E. histolytica*, Rotavirus	1
EAEC, Giardia, Rotavirus	3
**4. Pathogens**	
*Campylobacter*, EAEC, *E. histolytica*, Rotavirus	1

*EAEC, Enteroaggregative E.coli; EPEC, Enteropathogenic E.coli*.

## Discussion

The analysis for RV in stools collected during 2010–2015 from Nepal's largest birth cohort study, has shown a 7.6% prevalence of RV as the cause of infection in diarrheal episode stool samples. This observed prevalence is very similar in community based studies performed in India and other countries (13, 26). Many hospital-based studies found 30–40% acute gastroenteritis cases in children to be caused by RV. Many hospital-based studies found that 30–40% of acute gastroenteritis cases in children were caused by RV (18, 27–29). This shows that the prevalence of RV is higher in hospital-based studies compared to the community-based studies.

In our study, we detected RV in young children, mainly in the age from 7 to 12 months. Similar age distribution of RV infection was seen in previous epidemiological studies in Nepal, India as well as other Asian countries (17, 30–32). It has been shown that children are protected against severe RV diarrhea by maternal antibodies transferred during pregnancy or during the lactation period (33, 34). This could explain why children below 6 months of age have a lower risk of RV compared to those above 6 months of age.

RV is known as winter diarrhea but the occurrence varies from year to year and between geographical areas. In most studies from Nepal, the incidence peaks between January and March which is the coolest and driest period of the year (35). In our study the highest rates of rotavirus infection were seen in the winter month January. Our study confirms the findings of Uchida et al., with no cases in the month of August (36).

Molecular analysis of RV strain in our study showed that G2P[4] was the most prevalent strain in the Bhaktapur community followed by G1P[8], G12P[6], and G9P[8]. More than decade ago, in hospital-based RV studies in Nepal, have shown subtle changes in the genotype over time and geographical region. In year 2003 when the molecular study of Rotavirus begun in Nepal, by Uchida et al. (36), G1 was predominant, followed by G12 and G2. RV G9 strain was not detected in stool sample. The 3 years hospital-based study (November 2005 to October 2008) by Sherchand et al. (37), and the study by Ansari et al. (28), revealed G12 as an emerging genotype that was predominating in Nepal. In subsequent studies, G9 genotypes occurred in 2–6% of the cases (28, 36, 38). From 2014, a higher percentage of 31% of G9 was reported compared to previous studies in Nepal (18). A similar change has also been observed in the neighboring country India, where G9 increased from 2 to 10% in 2003–2007 to around 40% in 2013 (39). In this study, G9 in combination with P[4], P[6], and P[8] constitute 16% of the rotavirus positive samples. Currently, G9 genotype (particularly G9P[8]) is one of the six most common genotypes globally (along with G1P[8], G2P[4], G3P[8], G4P[8], and G12P[8]), causing ~90% of severe hospitalized RV cases (18, 40). The source of G9 and G12 genotypes in human is not known for certain but closely related G9 and G12 genotypes in pigs were reported, suggesting a potential porcine origin of these genotypes (41–43).

The study of Chawla-Sarkar et al. (12), covering a whole decade, showed uncommon combinations of RV strains like G12P[6], G1P[6], G12P[8], G2P[8] and G9P[6] which is consistent to our observation. Unusual RV genotypes have been reported to cause approximately 4.9% of RV diarrhea worldwide (40). The detection of uncommon genotypes and a high proportion of mixed infections indicate that the children probably acquire RV infections from various sources. This could lead to outbreaks of new strains globally. In a community, like in our study, humans and animals live in close proximity that can provide a perfect opportunity for dual, mixed infection encouraging rotavirus reassortment by interspecies or intraspecies transmission and the introduction of novel strains (44, 45). Due to unavailability of sequencing, we were not able to look into genomic changes of RV, antigenic drift and shift in this study.

In our study, the prevalence and pathogenicity of RV infection varied by age. We also found a various circulating strains in the community samples compared to samples collected from hospitals. It is therefore important to include community-based in addition to hospital-based surveillance studies to monitor the diversity of circulating RV strains. Recently, RV vaccines was introduced in the national immunization program in Nepal. We believe that it is important to follow a change in circulating pattern of RV strains after the introduction of the vaccine.

## Data Availability Statement

The raw data supporting the conclusions of this article will be made available by the authors, without undue reservation.

## Ethics Statement

Ethical approvals were received from the ethical board, Nepal Health Research council (NHRC), National IRB of Nepal and the Norweigen regional Ethical Committee for medical and health research (REK sør-øst B 42335 Norway). Written informed consent was obtained from the caretaker for all participants in the study.

## Author Contributions

All authors have contributed in planning the laboratory tests and also contributed in preparing and reviewing the manuscripts.

## Funding

This is part of self-sponsored Ph.D. project of JS.

## Conflict of Interest

The authors declare that the research was conducted in the absence of any commercial or financial relationships that could be construed as a potential conflict of interest.

## Publisher's Note

All claims expressed in this article are solely those of the authors and do not necessarily represent those of their affiliated organizations, or those of the publisher, the editors and the reviewers. Any product that may be evaluated in this article, or claim that may be made by its manufacturer, is not guaranteed or endorsed by the publisher.
